# Cardiovascular Disease among Syrian refugees: a descriptive study of patients in two Médecins Sans Frontières clinics in northern Lebanon

**DOI:** 10.1186/s13031-019-0217-x

**Published:** 2019-08-09

**Authors:** Philippa Boulle, Albane Sibourd-Baudry, Éimhín Ansbro, David Prieto Merino, Nadine Saleh, Rouba Karen Zeidan, Pablo Perel

**Affiliations:** 1 Médecins sans Frontières, Geneva, Switzerland; 2 Department of Noncommunicable Disease Epidemiology, London School of Hygiene and Tropical Medicine, London, UK; 3 Epidemiology and Biostatistics Department, Faculty of Public Health II, Lebanese University, Beirut, Lebanon; 4 Doctoral School of Science and Technology, Lebanese University, Beirut, Lebanon

**Keywords:** Cardiovascular disease, ASCVD, Refugee, Syria, Lebanon, Adherence, Humanitarian assistance, Secondary prevention

## Abstract

**Background:**

Literature on the burden and management of atherosclerotic cardiovascular disease (ASCVD) in humanitarian settings is limited. This study aimed to describe patient characteristics and explore both service use and use of recommended secondary prevention drugs in Syrian refugee patients with ASCVD attending two Médecins Sans Frontières (MSF) clinics in Lebanon.

**Methods:**

This study comprised a cross-sectional survey of ASCVD patients attending either MSF clinic over a four-week period in early 2017. Using descriptive statistics, we explored patient demographic characteristics, cardiovascular risk factors and assessed ASCVD secondary prevention medication prescription and patient adherence with a 7-day self-report scale. A retrospective study of routine clinical data explored workload and trends in patient loss to follow-up. We performed logistic regression modelling to explore risk factors for loss to follow-up.

**Results:**

We included 514 patients with ASCVD in the cross-sectional study, performed in 2017. Most (61.9%) were male and mean age was 60.4 years (95% CI, 59.6–61.3). Over half (58.8%) underwent revascularization and 26.1% had known cerebrovascular disease. ASCVD risk factors included 51.8% with diabetes and 72.2% with hypertension. While prescription (75.7 to 98.2%) and self-reported adherence rates (78.4 to 93.9%) for individual ASCVD secondary prevention drugs (ACE-inhibitor, statin and antiplatelet) were high, the *use* of all three was low at 41.3% (CI_95%_: 37.0–45.6). The 5-year retrospective cohort study (ending April 2017) identified 1286 patients with ASCVD and 16,618 related consultations (comprising 24% of all NCD consultations). Over one third (39.7%) of patients were lost to follow-up, with lower risk among men.

**Conclusions:**

The burden of ASCVD within MSF clinics in Lebanon is substantial. Although prescription and adherence of individual secondary prevention drugs is acceptable, *overall use* of the three recommended drugs is suboptimal. Loss to follow-up rates were high. Further studies are needed to evaluate innovative strategies to increase the use of the multiple recommended drugs, and to increase the retention of patients with ASCVD in the care system.

**Electronic supplementary material:**

The online version of this article (10.1186/s13031-019-0217-x) contains supplementary material, which is available to authorized users.

## Background

Non-communicable diseases (NCD) have become a major public health issue worldwide. Cardiovascular diseases (CVDs), cancer, diabetes and chronic lung diseases are the four key NCDs highlighted by WHO [1, 2]. Their burden continues to grow globally, but at a higher rate in low- and middle-income countries (LMICs). Among the 70% (40 million) of global deaths due to NCDs in 2016, CVDs were the leading cause (17.7 million) [2]. In addition, there are an estimated 422.7 million prevalent cases of CVD [1, 3]. Over three quarters of global CVD deaths occur in LMICs, which also bear the burden of most premature deaths (under the age of 70) [4].

Atherosclerotic cardiovascular diseases (ASCVDs), including coronary heart disease, stroke and peripheral vascular diseases, are the most common types of CVD, and patients with existing ASCVD are a population at particularly high risk of suffering a new CVD event [5].

Fortunately, there is high-quality evidence supporting the cost-effectiveness of pharmacological treatment for the secondary prevention of CVD events in patients with ASCVD, including β-blockers, angiotensin-converting-enzyme inhibitors (ACE-Is), statins, and aspirin. These interventions are recommended by all major international guidelines [6–8]. However, the use of this pharmacological treatment is worryingly low worldwide and remains a critical issue in LMICs where up to 80% of ASCVD patients do not have access to even one of the recommended drugs [9, 10].

As the global burden increases, medical humanitarian organisations, such as Médecins sans Frontières (MSF), are increasingly faced with patients needing care for NCDs, including those with ASCVD. While there is growing evidence on the burden and gaps in access to care for NCDs in humanitarian crisis settings, very little has been published on the cost effective management of NCDs in general, and still less on the management of ASCVD, in humanitarian settings [11–15]. This is a significant gap, especially given that limited evidence shows that crises may actually increase CVD mortality, morbidity and risk factors [12, 16–18]. Commentators have noted the challenges that exist around identifying and treating ASCVD even in high-income, stable settings with ready access to medications and diagnostics [19].

It may be more challenging still in crises, given the potential health system disruption, the lack of pre-crisis capacity or access to ASCVD medical management, diagnostics and interventions in many LMICs (particularly at primary care level), and humanitarian actors’ relative inexperience in managing ASCVD. The MSF approach in many settings has been to provide a regular supply of good quality, secondary prevention medications at primary care level along with adherence support for patients with a self-reported history of ASCVD but, thus far, ASCVD cohorts have not been characterised and this approach has not been evaluated.

Over six million Syrians have left their country since the start of the Syrian conflict in 2011 [20]. Neighbouring Lebanon has a national population of around 4 million, and currently hosts an estimated 1.5 million Syrian refugees, of whom just over 1 million are registered with the United Nations High Commissioner for Refugees (UNHCR) [21–23]. NCDs are highly prevalent among the Syrian population. Prior to the conflict, 77% of all deaths in Syria were caused by NCDs, with CVD alone responsible for 44% [24]. A 2014 survey of Syrian refugees in Lebanon found that 50.4% of surveyed households reported at least one member with a chronic condition, including cardiovascular disease in 10.8% of the households [25].

The Lebanese Ministry of Public Health (MoPH) and UNHCR coordinate a complex group of providers to serve Syrian refugees, and UNHCR-registered refugees can access a network of primary care centres [15]. However, they are required to make subsidised co-payments for consultations, medications and referrals for hospital treatment; accessibility varies between areas; and access to hospital-based services is very limited [15, 26]. Registration of Syrian refugees by UNHCR was suspended in May 2015, following Lebanese government instruction, and unregistered refugees are limited to attending facilities funded by private donors or humanitarian organisations, such as MSF [15, 27].

MSF has been providing free-of-charge healthcare to Syrian refugees and the vulnerable host community in Lebanon, in parallel to the MOPH system, since early 2012 in both northern Lebanon and in the Bekaa valley. At the time of the study, MSF was providing integrated primary health care (PHC) with general PHC consultations, sexual and reproductive health, mental health and NCD outpatient care, including secondary prevention for patients with ASCVD.

This study aimed to describe the magnitude of the burden of ASCVD in patients seeking care in two MSF clinics in northern Lebanon, and the characteristics and pattern of care of these patients.

Specifically, our objectives were to describe patients with ASCVD in terms of:

1. Frequency of the different atherosclerotic cardiovascular conditions
2. Demographic characteristics
3. Medication prescription, adherence and use
4. Pattern of follow-up

Our final goal was to identify current challenges in the model of care in order to find ways to improve the management of patients with ASCVD in the humanitarian context.

## Method

### Study design

This is a descriptive study with two components: a cross-sectional survey and a retrospective cohort study.

### Setting

The study took place in two MSF clinics in northern Lebanon. Dar Al Zahara (DAZ) clinic, open since 2012, is located within a hospital in Tripoli town, North Lebanon governorate. Abdeh clinic, open since April 2015, is situated in Abdeh village, in Akkar governorate north of Tripoli. In both, MSF is providing integrated primary health care to Syrian refugees and vulnerable Lebanese, including NCD care for the following chronic conditions: hypertension, diabetes, cardiovascular disease, epilepsy, hypothyroidism and chronic respiratory disorders. At the time of the study NCD care was offered only to non-Lebanese nationals as Lebanese patients could access such care through a national NCD program.

For ASCVD patients (as for other NCD patients), the offered services included consultations, the provision of essential medications, access to basic investigations, patient education and when necessary, specialist referral. There was an appointment system for patient consultations, but no system to remind patients of upcoming appointments. Care was provided by non-specialist general practitioners (GPs) and nurses, supported by an international specialist doctor. Nurses saw all patients for a check of vital signs, and to organise medication refill if patients fit specific criteria of stability. Patients were then referred to the doctor in case of complications or for every third visit. Laboratory tests were performed on site through point-of-care tests in Abdeh clinic, and in the hospital laboratory in DAZ clinic, according to a rationalised schedule of routine investigations. Patient education, including on hypertension and ASCVD, was provided in the waiting areas by health promotion staff, and individually to patients by trained nurses. Medications and related instructions were given by a pharmacy dispenser nurse. All information collected during consultations was recorded on individual paper files kept at the clinic, and then routinely entered into an MSF database in a disaggregated manner.

Consultation frequency was usually monthly, with consultations of two-monthly frequency being implemented once patients had reached target blood pressure. Patients were treated according to MSF protocols, which were adapted for the local context from leading international guidelines. For the secondary prevention of ASCVD, they involved the concomitant use of an antiplatelet agent and statin, and potentially ACE inhibitors and β-blockers. Relevant extracts from MSF’s Integrated Clinical Pathway for Patients at High Cardiovascular Risk are available in a supplementary file [see Additional file 1].

### Participants, data sources and collection

#### Cross-sectional survey

The cross-sectional survey aimed to describe the characteristics of ASCVD patients.

Participants: The study inclusion criteria were patients aged 18 years and older with established atherosclerotic cardiovascular disease [history of coronary heart disease (CHD), cerebrovascular disease, or peripheral vascular disease (PVD)] enrolled in the NCD cohort of DAZ and Abdeh MSF clinics in northern Lebanon, attending the clinic during a four-week period between March 8th and April 5th 2017.

Variables collected:

1. Demographic and socio-economic characteristics (age, gender, country of origin, refugee status, address, occupation) as well as date of enrolment in MSF NCD cohort,
2. ASCVD diagnoses,
3. Cardiovascular risk factors,
4. Cardiovascular secondary prevention medications prescribed (including date of initiation and current daily dose),
5. Self-reported adherence, and
6. Causes of non-adherence.

ASCVD diagnoses were grouped as (a) Coronary heart disease [myocardial infarction (MI) and angina], (b) cerebrovascular disease [stroke, transient ischaemic attack (TIA)], (c) PVD, (d) previous revascularization [percutaneous coronary angioplasty (PTCA) and coronary artery bypass graft (CABG)]. These were largely based on self-report, although a limited number of refugees arrived in Lebanon with medical records confirming their prior ASCVD diagnoses and patients diagnosed since arrival in Lebanon usually had their diagnoses medically confirmed.

Cardiovascular risk factors collected were: comorbid diabetes, hypertension and current smoking. These were primarily based on self-report, unless the diabetes or hypertension had been newly diagnosed by MSF staff.

Self-reported adherence was assessed using a purpose-designed tool, asking patients the number of days they had taken the relevant drugs as prescribed (the correct number of pills per day) during the previous 7 days, as has been done in other studies [28]. This question was asked for each class of drug they were prescribed for secondary prevention of ASCVD (ACE-I, statin, β-blocker, antiplatelet agent). The patient was considered adherent, for each drug, if the correct number of pills was taken for 5 days or more.

The reasons for non-adherence to any of the recommended drugs were systematically explored using a pre-defined checklist and were based on self-report. The checklist was adapted following a total of eighteen pre-tests, which were performed in both clinics. The patient was asked to volunteer a reason for non-adherence and the nurse then applied a checklist category or detailed it under “other”. The categories were: on doctor’s instructions, forgetfulness, unable to attend clinic, feeling unwell, side effects due to the drug, choosing to take the drug not as prescribed, taking too many drugs, indifference, don’t know (e.g. family member handling drugs), other. An additional reason, “did not understand prescription”, was added after the study commenced since staff frequently encountered this response. We also explored frequency of drug “use”, aiming to capture both adherence by clinicians to prescribing guidelines (via clinical records) and adherence by the patient to taking medications as prescribed (via self-report).

Data sources and collection:

A case report form (CRF) was designed to collect the required information. Every eligible patient seeking care at one of the two MSF clinics during the study period was invited to participate in the study, and every consenting patient signed an informed consent form.

The 4-week period was chosen to maximise participation as the majority of the ASCVD patients were still on a monthly follow-up routine. We expected that approximately 500 patients would fulfil the eligibility criteria. With this sample size, the expected precision for adherence lay between 4.29 and 3.13% for a true prevalence between 60 and 85%.

Each clinic had its own team in charge of implementing the CRF. The NCD nurse was in charge of identifying eligible patients and obtaining informed consent. For consenting patients, the GP filled the ASCVD history and prescription parts of the CRF and referred the patient to the Patient Support Education and Counselling (PSEC) nurse after the consultation. The PSEC nurse recorded the demographic and adherence information on the CRF.

All clinical MSF staff involved in the cross-sectional part of the study were trained on their specific roles and the process for filling the CRF was the same in the two clinics. A pilot test was performed on 8 to 10 patients in each clinic and the CRF was adapted according to the feedback received.

### Retrospective cohort study

The objective of the retrospective cohort study was to analyse the burden of ASCVD in patients attending for medical care at MSF clinics in northern Lebanon, as well as the pattern of follow up visits.

#### Participants

The study inclusion criteria were patients aged 18 years and older with established atherosclerotic cardiovascular disease (history of CHD, cerebrovascular disease, or PVD) enrolled in the NCD cohort of DAZ and Abdeh MSF clinics in northern Lebanon and attending either clinic since its opening until the end of April 2017.

#### Data, sources, and collection

Data regarding visit dates, type (planned/unplanned) and frequency for all ASCVD patients since the opening of each clinic were extracted from the MSF NCD database containing routinely collected data. These data were cleaned before analysis.

### Statistical analysis methods

Categorical variables were described with proportions and continuous variables were summarized with means overall and per clinic. We calculated confidence intervals for the overall estimates and for the clinic level means and proportions.

We explored whether some baseline demographics (sex, age, resident permit, Arabic literacy), time from first visit and clinical factors (ASCVD diagnosis, risk factors) were associated with non-adherence. We coded the factors into binary variables and we used Fisher’s exact tests to compare the proportion of adherent patients between the two categories of each variable.

Patients who were prescribed an ACE-I, statin, β-blocker or antiplatelet agent for the first time at the survey visit were not included in this analysis (unless they were new patients). This was due to a concern that the prescription of such drugs to existing patients at that visit may have been a “corrective measure” taken by the GPs who were responsible for filling out the section of the CRF on prescribed medicines. Additionally, self-reported adherence for angiotensin II receptor blockers (ARBs) was not asked. For the self-adherence analysis, we assumed that adherence to ARBs would have been the same as that for ACE-Is.

With the dates of visits from the retrospective cohort we calculated the number of visits and estimated distribution of delay between visits. We defined a “loss to follow up” (LTFU) episode when a patient had no contact with the clinic for 100 or more consecutive days. We calculated the distribution of LTFU episodes in the patients and we used a logistic regression model to analyse whether sex and age at baseline, only, were associated with the risk of having at least one loss to follow up episode. The model was adjusted for time since enrolment.

### Ethical approval

This study received ethical approval from the MSF ethics review board and from the Lebanese University ethical review board.

Our findings are reported here according to STROBE statement guidelines for observational studies [29].

## Results

### Cross-sectional study

A total of 545 eligible patients with ASCVD were identified during the 4-week period, of whom 94.3% (514) accepted to participate (238 in Abdeh clinic and 276 in DAZ clinic). 31 patients refused to participate, of whom 77% were in Abdeh clinic (*n* = 24). A third did not give any reason for their decision, 23% had no time to spare, 16% did not feel comfortable participating in a study and a quarter said they did not need/want any improvement in the care they received in MSF clinics (the study was presented as a step for MSF to improve the quality of care being provided).

#### Patient characteristics

Of the 514 patients who participated, 98.8% of them were Syrian refugees, among whom 98.2% were registered with UNHCR. Table 1 shows the main characteristics of the patients.

**Table 1:** Characteristics of ASCVD patients taking part in cross-sectional survey in MSF clinics, north Lebanon

Characteristics	Overall [95% CI]^a^	Abdeh clinic [95% CI]	DAZ clinic [95% CI]
Demographic			
Age (mean)	60.4 [59.6, 61.3]	60.8 [59.6, 62.1]	60.1 [58.9, 61.3]
Gender (male)	61.9 [57.5, 66.1]	66.0 [59.5, 71.9]	58.3 [52.3, 64.2]
Resident permit	27.2 [23.5, 31.3]	20.2 [15.4, 25.9]	33.3 [27.9, 39.3]
Reads Arabic	58.9 [54.5, 63.2]	56.7 [50.2, 63.1]	60.9 [54.8, 66.6]
ASCVD history			
Coronary heart disease	31.5 [27.6, 35.8]	13.0 [9.2, 18.1]	47.5 [41.5, 53.5]
Cerebrovascular disease	26.1 [22.4, 30.1]	27.7 [22.2, 34]	24.6 [19.8, 30.2]
Peripheral vascular disease	1.8 [0.9, 3.4]	1.7 [0.5, 4.5]	1.8 [0.7, 4.4]
Revascularisation	58.8 [54.4, 63]	66.0 [59.5, 71.9]	52.5 [46.5, 58.5]
Risk factors			
Diabetes	51.8 [47.3, 56.1]	49.2 [42.7, 55.7]	54.0 [47.9, 59.9]
Smoking	39.7 [35.5, 44.1]	38.2 [32.1, 44.8]	40.9 [35.1, 47.0]
Hypertension	72.2 [68.1, 76.0]	66.4 [60.0, 72.3]	77.2 [71.7, 81.9]
All 3 risk factors	12.1 [9.4, 15.3]	9.2 [6.0, 13.8]	14.5 [10.7, 19.3]

^a^Data are presented as proportions or means with 95% Confidence Intervals

Demographic characteristics of the ASCVD patients were comparable between the 2 clinics: mean age of 60.4 (95% CI: 59.6–61.3) years old, majority male (61.9%) and over half literate in Arabic (58.9%). The proportion of patients with a valid resident permit was significantly higher in DAZ than in Abdeh, but still low in both locations, at 33.3 and 20.2% respectively.

With respect to ASCVD history, more than half of the patients (58.5%) had at least one revascularisation (CABG or PTCA), with a higher proportion in Abdeh (66%) compared to DAZ (52.5%). The reported prevalence of CHD was very different between the 2 clinics with only 13% in Abdeh but 47.5% in DAZ. A quarter (26.1%) had a diagnosis of cerebrovascular disease, while only 1.8% had been diagnosed with PVD. About 1 in 5 of the recruited patients (18%) had more than one ASCVD diagnosis.

Regarding cardiovascular risk factors, about half of the patients (51.6%) had diabetes. A majority had hypertension (72.2%); the proportion was higher in DAZ (77.2%) than in Abdeh (66%). Almost 40% of the patients were current smokers. About 1 in 10 of the recruited patients (12.1%) reported all 3 risk factors.

#### Prescription

A high proportion of patients were prescribed treatment for ASCVD secondary prevention as recommended in the MSF Guidelines (Table 2). Almost all patients were prescribed statin (98.2%) and antiplatelet (97.3%) medication. Over three quarters of the patients were prescribed β-blockers (79.1%) or ACE-I or ARBs. (75.7%). 83.2% of the patients were prescribed three recommended drugs: statin, antiplatelet and at least one anti-hypertensive drug (ACE-I, ARB or β-blockers), while 73.5% were prescribed all three recommended drugs among ACE-I/ARB, statin and antiplatelet.

**Table 2:** Prescribed ASCVD related treatment

Drug	Overall [95% CI]	Abdeh clinic [95% CI]	DAZ clinic [95% CI]
ACE-I or ARB	75.7 [71.7, 79.3]	80.3 [74.64, 85.1]	71.8 [66.0, 77.0]
Statin	98.2 [96.5, 99.1]	95.6 [93.1, 98.4]	99.6 [97.7, 100]
Antiplatelet	97.3 [95.3, 98.4]	95.3 [91.5, 97.5]	98.9 [96.6, 99.7]
Β-blocker (BB)	79.1 [75.2, 82.5]	73.8 [67.6, 79.2]	83.5 [78.5, 87.6]
Statin, antiplatelet, ACE-I/ARB	73.5 [69.4, 77.3]	76.4 [70.3, 81.6]	71.1 [65.2, 76.3]
Statin, antiplatelet, ACE-I/ARB/BB	83.2 [79.6, 86.3]	79.8 [74.0, 84.7]	86.1 [81.3, 89.8]

Among patients who were not prescribed all the recommended drugs, the main reason for not prescribing ACE-I/ARB or β-blocker, according to prescribing clinicians, was because there was “no need” for it, in 38.7 and 89.9% of cases respectively. Occurrence of side effects represented 31.2% of the cases of non-prescription of ACE-I/ARBs but only 1.1% for β-blockers.

#### Self-reported adherence

Self-reported adherence rates are presented in Table 3. Self-reported adherence to individual drugs was in general high but disparate, with lower rates in DAZ clinic. Self-reported adherence to antiplatelet treatment was very high (93.9%), as it was for β-blocker treatment (90.8%). Self-reported adherence for statins was high in Abdeh (91.2%) but lower in DAZ (67.7%). The same trend was observed for ACE-I, with 90.8% of patients in Abdeh reporting a good level of adherence compared with 75.2% in DAZ. For patients who were prescribed 3 recommended drugs including one anti-hypertensive drug, self-reported adherence was 85.2% in Abdeh and 57.3% in DAZ.

**Table 3:** Self-reported adherence among ASCVD patients taking part in cross-sectional study in MSF clinics, north Lebanon

Drug	Overall (%, 95CI)	Abdeh clinic (%, 95 CI)	DAZ clinic (%, 95 CI)
ACE-I	83.8 [79.1, 87.6]	90.8 [85.2, 94.5]	75.2 [67.1, 81.9]
Statin	78.4 [74.4, 81.9]	91.2 [86.5, 94.4]	67.7 [61.7, 73.1]
Antiplatelet	93.9 [91.3, 95.8]	95.6 [91.7, 97.7]	92.5 [88.5, 95.2]
β—blocker	90.8 [87.4, 93.4]	94.1 [75.2, 87.4]	88.3 [83.2, 92.1]
Statin, antiplatelet, ACE-I	69.5 [64.0, 74.6]	82.0 [75.2, 87.4]	54.4 [45.7, 62.8]
Statin, antiplatelet, ACE-I/BB	72.6 [67.2, 77.5]	85.2 [78.7, 90.0]	57.3 [48.5, 65.5]

Table 4 shows the causes of non-adherence for each drug that patients reported having taken for four days or fewer during the previous week, or at a different dose to the one prescribed (a different number of pills).

**Table 4:** Causes of non-adherence (all patients)

Ranking	Overall (*N* = 228)	ACE-I (*N* = 53)	Statin (*N* = 108)	Antiplatelet (*N* = 30)	BB (*N* = 37)
1st	Did not understand prescription (46%)	Did not understand prescription (47%)	Did not understand prescription (67%)	Doctor’s request (20%)	Did not understand prescription (22%)
2nd	Patient’s decision (11%)	Patient’s decision (17%)	Side effects (6%)	Forgot (16%), Side-effects, (16%)	Patient’s decision (16%)
3rd	Side-effects (7%), Doctor’s request (7%)	Doctor’s request (6%), other (6%)	Patient’s decision (5%), MSF-stock ruptures (5%)	Patient’s decision (11%)	Side effects (14%)

Overall (Table 4), the main reason for non-adherence to one of the drugs was that patients did not understand the prescription in terms of dose (the number of pills they had to take each day), and hence did not take the drug “as prescribed” (46%). This was especially true for statins (67%) and ACE-Is (47%) but it was not a reason for non-adherence to antiplatelets. The second most frequent cause of non-adherence was patient decision (11%), followed by experiencing side effects or following doctor’s request (7%). This last reason was the leading one for low self-reported adherence to antiplatelets (20%).

Non-adherence to statins was associated with having a first consultation in an MSF clinic more than two years ago (*p* = 0.008) and having a history of CHD (*p* = 0.002), and revascularisation was associated with increased adherence to statins (*p* = 0.006). No strong evidence for an association was found between demographics or other clinical factors and adherence to any other treatment.

Overall the combined self-reported adherence to prescription of at least three drugs indicated for ASCVD secondary prevention (statin, antiplatelet and one anti-hypertensive drug) was low at 43.4% (CI_95%_: 39.1–47.8) of the surveyed patients, with a marked difference between the two clinics: 60.5% (CI_95%_: 54.0–66.7) in Abdeh clinic and only 28.6% (CI_95%_: 23.4–34.4) in DAZ clinic.

### Retrospective cohort study

#### Cardiovascular disease burden

From the opening of each of the two clinics until the end of April 2017, the total number of ASCVD patients seen was 1286. Of a total of 16,686 ASCVD consultations, 92.3% were follow-up consultations (*n* = 15398). The consultations for patients with ASCVD represented 24% of the NCD workload (of 69,010 total NCD consultations during this period). Over 50% of the ASCVD patients had other NCD comorbidities for which they were also reviewed during their consultations.

#### Follow-up pattern

**Fig. 1:**
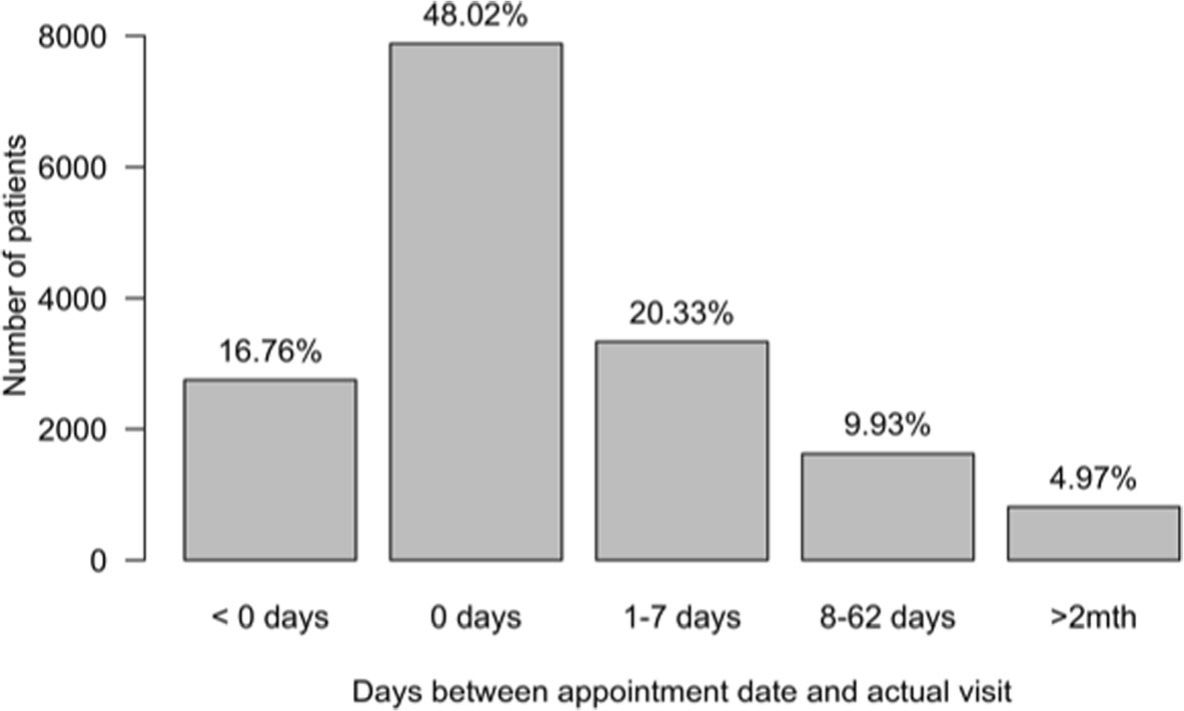
Distribution of delay between appointment and visit date

Table 5 presents treatment interruption and loss to follow up patterns while Fig. 1 shows frequency of follow up and patients' adherence to planned appointments. In the multivariable logistic regression analysis, the only variable associated with LTFU was sex; men had reduced odds of having at least one LTFU episode (OR = 0.72, 95% CI 0.56 to 0.92, *p*-value = 0.0099) compared to women.

**Table 5:** Patterns of loss to follow up or treatment interruption, follow-up frequency and adherence to planned appointments

Loss to follow up	Total (*n* = 1,286)
No return after 1st visit	138 (10.7%)
One or more treatment interruption	743 (58%)
More than one interruption	157 (12.2%)
Lost to follow up at end of study period	510 (39.7%)

## Discussion

### Main findings and comparison with previous studies

Our study shows that a high proportion of clinic workload (24%) is related to patients with a diagnosis of ASCVD among those being treated for NCDs in MSF clinics in northern Lebanon. The mean age (60 years) of the patients who participated in the cross-sectional survey is consistent with other studies that found higher rates of CVD among Syrian refugees in Lebanon older than 60 years old and with the fact that CVD prevalence increases with age [25, 27, 30]. A higher proportion of men among ASCVD patients reflects the increased risk of ASCVD morbidity and mortality in men globally and specifically in Syria [31]. This correlates with results from the Global Health Data Exchange for Syria according to which DALYs associated with Ischemic Artery Diseases and stroke represent 35.5% for men, compared to 24% of women aged 50–69 years old. This holds true for this cohort despite the fact that among Syrian refugees in Lebanon there are more adult women than men in the age ranges 18–59 men and 60+ [22].

In terms of ASCVD history, revascularisation was the most frequently reported manifestation of CVD, with more than half (58.8%) of the ASCVD patients having undergone such a procedure, reflective of the high level of medical care available in Lebanon and in Syria prior to the war. However, fewer patients (31.5%) had a diagnosis of CHD (here defined as angina or MI). The apparent difference in the prevalence of CHD between the two clinics (13% in Abdeh and 47.5% in DAZ) may be due to differences in reporting of medical history. It appeared that medical teams were not routinely recording a diagnosis of CHD for patients with previous revascularisation, and this was more common in Abdeh clinic, where only 3% of patients with previous revascularisation were documented as having CHD compared to 40% in DAZ clinic.

The high prevalence of risk factors such as hypertension and diabetes in ASCVD patients is as expected and has been well documented in similar populations [32–34]. Abdul Rahim et al. reported very similar and increasing rates of smoking in the Arab world generally [35].

MSF protocols for the secondary prevention of ASCVD events were generally well followed and treatment prescription was satisfactory, with recommended drugs being prescribed to at least three quarters of the patients. According to the programme doctors, the main reason for not prescribing β-blockers or ACE-I/ARB (the two least prescribed classes of drugs) was that there was “no need”.

It is interesting to note that having the doctors fill the prescription part of the CRF had a positive side-effect. For 12.3% of patients they corrected the prescription immediately by adding recommended drugs that were found to be missing (either never prescribed or disrupted prescription for no apparent specific reason). There was clearly room for improvement in prescribing, but this act of drug review served to increase prescription fidelity and shows that the clinicians may benefit from reminders to review prescriptions from one follow-up consultation to the next, even when there is a high workload. Similarly, performing periodic prescribing audits in chronic care programmes may help to combat prescriber fatigue and inertia.

Overall, patients reported good adherence to their ASCVD treatment. This finding is consistent with those from a study of adherence to NCD medication of Syrian refugees in Lebanon [36], although a systematic review on adherence to CVD medication in resource-limited settings reported overall adherence at a much lower rate of 57.5% [37].

We found strong evidence of an association for non-adherence to statins with first consultation in MSF clinic taking place more than two years ago and with a CHD diagnosis. By contrast, we found strong evidence of an association between previous revascularisation and *increased* adherence. Although reasons for these were not explored, it is plausible that the latter group of patients had a better understanding of the need for statin use and thus greater motivation for adherence.

Although prescription and self-reported adherence were acceptable, the combination of the two resulted in a low overall use of the recommended drugs (43.4%). Overall use was much lower in DAZ clinic (28.6%) than in Abdeh clinic (60.5%), resulting from a lower self-reported adherence in DAZ clinic.

Analyses of the apparent adherence disparity between the clinics revealed that the main causes were provider-driven (due to drug shortages leading to short-term changes in dosing, which were inadequately explained to patients), and could therefore be mitigated. On questioning, many patients appeared to have poorly understood their prescription: they had taken the drugs for at least five days during the previous week, but not the appropriate number of pills, anecdotally showing genuine surprise when this was explained to them. This was especially true for statins (accounting for 70% of low adherence cases) as well as for ACE-I. This issue correlated with a change in prescription due to drug shortages that happened a month prior to the study, which led to either a change in the pill strength (simvastatin 20 mg to simvastatin 10 mg), a change between ACE-I drugs (enalapril to captopril), or change of class of drug (ARB instead of ACE-I), in many cases necessitating a change in the daily number of pills taken. In some cases, complete stock outs occurred leading to treatment interruptions.

The second most frequent cause for low adherence was a patient’s decision not to take certain drugs either for the whole week or on a certain number of days. These two reasons highlight the importance of patient support and education in the care process, the value of patient-centeredness, shared decision making, understanding the patient’s beliefs and concerns regarding medications, and tailoring advice and support to each patient [38].

Patients partially followed their appointment schedule: just under half of the consultations took place on the appointment date and an additional 20.3% occurred within the following week. However, having more than half of patients not attend at their scheduled appointment time has a direct implication for work organisation, teams’ workload and quality of care. Improving (and simplifying) the appointment system - such as with a patient reminder system - may prove beneficial. Two-monthly appointments were recommended in the project for stable patients, with potential benefits of decreased clinic workload and burden of attendance for patients. The proportion of patients on this schedule was relatively low but increasing. It was higher in DAZ (22.7%) than in Abdeh (6%), likely due to Abdeh clinic having opened more recently, meaning that patients attending this clinic had had less time to stabilize.

More than half of the patients had at least one episode of loss to follow-up. The process for tracking these patients properly may need to be reinforced, partly to help with understanding contributory causes and identifying those that are modifiable.

### Strength and limitations

To our knowledge this is the first study to evaluate the burden of patients with ASCVD and describe their management with a focus on use of recommended medications in humanitarian settings. Most of the data analysed for this study were collected during the cross-sectional component, which lasted one month. Completeness and reliability were therefore increased compared to routinely collected data. The participation rate was high at 94.3% and so selection bias is likely to be minimal.

The data presented here are only representative of Syrian refugees with known ASCVD seeking care in two MSF clinics in Northern Lebanon, and it might not be representative of the overall ASCVD burden among Syrians living in Lebanon.

The main biases that could affect the results of this study are recall bias and acquiescence bias that may have led to an overestimated self-reported adherence. There is a broad range of validated adherence surveys, specifying timeframes from 1 day to 12 months [39]. We tried to minimise potential recall bias by asking about adherence only over the preceding 7 days. This tool has been used in other studies but is not externally validated [28]. Adherence questions were asked by the PSEC nurses, who are part of the regular MSF care team. Until then, they were little involved in the management of ASCVD patients. However, half of the participants also had diabetes and PSEC nurses play an active role in the care of diabetic patients. The existing and ongoing care relationship between the PSEC nurses and some patients could have led to response bias for some of the patients.

Reporting of the number and type of ASCVD events could also be affected by recall bias, especially since many patients arrived from Syria without any medical records. However, validation studies using a variety of tools over the last decade have shown a relatively robust sensitivity of 78–98% for self-reported MI [40].

The adherence question was asked for ACE-I but not for ARB. For the analyses of the overall adherence and use of drugs, we assumed that the adherence to ARB would have been the same as the adherence to ACE-I.

For the retrospective study we reported the data included in the existing information system and there was a degree of missing data. However, there were no missing data in the cross-sectional survey.

Regarding patients lost to follow-up, some may have been considered lost to follow-up at the time of analysis but might have returned to care at a later date. This study has not explored the characteristics of the subpopulation that had unplanned periods of loss to follow up or definitively left the programme.

### Implications for research

This research yielded some results that could be interesting to investigate further. Among the main causes of non-adherence, we identified the lack of understanding by patients of the prescription of multiple drugs. The use of a fixed dose combination drug (a polypill) including a statin, aspirin, and at least one anti-hypertensive has been shown to be effective in research studies and may be a simple strategy to increase adherence in humanitarian settings [41]. Additionally, exploring patient perceptions, experiences and understanding of statins and their medical importance to coronary heart disease could help us to understand why patients were less likely to be adherent to statins than to other drugs. Further studies to explore the reasons for loss to follow up would be important to better understand the patient and clinic level barriers in order to develop specific strategies to tackle these.

Overall, it is important to design and evaluate simplified models of care to help increase prescription rates, adherence, and overall use of cardiovascular secondary prevention medications and to ensure regular follow-up of patients with ASCVD, while balancing with clinic capacity. Potential components of these simplified models could include decreased consultation frequency for stable patients as well as an enhanced community involvement, especially in the context of protracted humanitarian crises. Community health programmes have been reported to positively support vulnerable populations and refugees to access health care [9, 36]. Because of MSF’s substantial experience in managing patients with HIV/AIDS (also necessitating chronic care) in humanitarian settings it could be interesting to adapt some of the lessons learnt from these models to patients with ASCVD.

Finally, developing an information system that could better monitor the impacts of programmatic changes could be a powerful tool to support informed decision-making.

## Conclusion

The burden of CVD and other NCDs is increasing globally and even more rapidly in LMICs. This epidemiological transition has a direct impact on the needs of crisis-affected populations and hence on the care that needs to be provided by aid organisations. Providing adequate CVD care in limited-resource settings remains a challenge. MSF has developed a model to provide NCD care in high-workload humanitarian settings, utilising non-specialist general practitioners, with tasks shifted from doctors to nurses, and using a rationalised list of standardized drugs. Our study showed overall acceptable results in these two clinics in Lebanon: patients were following their care recommendations with reasonable follow-up rates and adherence to treatment, and prescriptions aligned with the protocols. However, MSF is working to continue to improve, refine and evaluate effective and sustainable NCD care models for humanitarian settings.

## Additional file

Additional file 1: **Annex 1.** Extracts from “Integrated Clinical Pathway for Patients at High Cardiovascular Risk”, MSF. (DOCX 82 kb)

## Data Availability

The datasets used and/or analysed during the current study are available from Médecins sans Frontières- Operating Centre Geneva on reasonable request.

## Abbreviations

ACE-I: Angiotensin Converting Enzyme Inhibitor
ARB: Angiotensin Receptor II Blocker
ASCVD: Atherosclerotic Cardiovascular Disease
BB: β-blocker
CABG: Coronary Artery Bypass Graft
CHD: Coronary Heart Disease
CI: Confidence Interval
CRF: Case Report Form
CVD: Cardiovascular Disease
DALYs: Disability Adjusted Life Years
DAZ: Dar Al Zahara
GBD: Global Burden of Disease
GP: General Practitioner
HIV/AIDS: Human Immunodeficiency Virus/Autoimmune Deficiency Syndrome
LMICs: Low- and Middle-Income Countries
LTFU: Lost to Follow Up
MOPH: Ministry of Public Health
MSF: Médecins sans Frontières
NCD: Noncommunicable Disease
NGO: Non-governmental Organisation
PHC: Primary Health Centre
PSEC: Patient Support Education and Counselling
PTCA: Percutaneous Transluminal Coronary Angioplasty
PVD: Peripheral Vascular Disease
STROBE: Strengthening the Reporting of Observational Studies in Epidemiology
TIA: Transient Ischaemic Attack
UNHCR: United Nations High Commissioner for Refugees
WHO: World Health Organization
