# Supplementary material for: Cardiovascular Disease among Syrian refugees: a descriptive study of patients in two Médecins Sans Frontières clinics in northern Lebanon
Source: Confl Health. 2019 Aug 9;13:37. doi: 10.1186/s13031-019-0217-x (PMC6688221; doi:10.1186/s13031-019-0217-x)
Supplement: Supplementary file 1 — Annex 1. Extracts from “Integrated Clinical Pathway for Patients at High Cardiovascular Risk”, MSF. (DOCX 82 kb) [file 13031_2019_217_MOESM1_ESM.docx]

**Annex 1: Extracts from “Integrated Clinical Pathway for Patients at High Cardiovascular Risk”, MSF**

Patients with atherosclerotic cardiovascular disease (ASCVD) technical note (p.17)

Patients with ASCVD (angina pectoris, coronary heart disease, myocardial infarction, stroke, peripheral vascular disease or after coronary revascularization or carotid endarterectomy) are at very high risk of developing recurrent cardiovascular events.

 ALL THESE PATIENTS SHOULD RECEIVE

1)        75-100 mg aspirin per day unless there are clear contraindications

*Aspirin contraindications*:

i)     Allergy to aspirin

ii)     High risk of bleeding

Defined as history of complicated peptic ulcer disease or at least two of the following factors (age> 60 years old, high dose of NSAID, previous uncomplicated ulcer, concurrent use of corticosteroid or anticoagulants).

Patients with ASCVD and contraindications to aspirin should receive Clopidogrel (75mg/day)

Patients receiving a bare-metal stent or drug-eluting stent during PCI for ACS should receive clopidogrel 75 mg daily with low dose aspirin (75 to 100 mg daily) and continue only with aspirin after 12 months.

 2)        Patients with ASCVD should receive statins (see cholesterol notes for doses)

Patients with history of myocardial infarction or coronary heart disease and left ventricular dysfunction should receive β-Blockers and ACE-inhibitors regardless of their blood pressure (see blood pressure technical notes for doses)
